# HPV16 Intratypic Variants in Head and Neck Cancers: A North American Perspective

**DOI:** 10.3390/v15122411

**Published:** 2023-12-12

**Authors:** Steven F. Gameiro, Mikhail Y. Salnikov, Peter Y. F. Zeng, John W. Barrett, Anthony C. Nichols, Joe S. Mymryk

**Affiliations:** 1 Department of Microbiology and Immunology, The University of Western Ontario, London, ON N6A 3K7, Canada; gameiros@mcmaster.ca (S.F.G.); msalnik@uwo.ca (M.Y.S.); 2 Department of Pathology and Laboratory Medicine, The University of Western Ontario, London, ON N6A 5C1, Canada; peter.zeng@lhsc.on.ca (P.Y.F.Z.); anthony.nichols@lhsc.on.ca (A.C.N.); 3 Department of Otolaryngology, The University of Western Ontario, London, ON N6A 5W9, Canada; john.barrett@lhsc.on.ca; 4 Department of Oncology, The University of Western Ontario, London, ON N6A 5W9, Canada; 5 London Regional Cancer Program, Lawson Health Research Institute, London, ON N6A 5W9, Canada

**Keywords:** human papillomavirus, intratypic variants, E6, TCGA, head and neck squamous cell carcinoma

## Abstract

Human papillomavirus (HPV) is the major causative agent for cervical and many head and neck cancers (HNCs). HPVs randomly acquire single nucleotide polymorphisms (SNPs) that may become established via positive selection. Within an HPV type, viral isolates differing by <2% in the L1 region are termed “variants” and classified based on combinations of SNPs. Studies in cervical cancer demonstrate clear differences between HPV16 intratypic variants in terms of persistence of infection, tumor histology, cancer risk, and death. Much less is known about the frequency of HPV16 variants in HNC, and their effects on clinical outcomes. We combined HPV16 positive (HPV16^+^) HNC samples from a local Southwestern Ontario, Canada cohort with those from the Cancer Genome Atlas to create a larger North American cohort of 149 cases with clinical data and determined the distribution of intratypic variants and their impact on clinical outcomes. Most isolates were lineage A, sublineage A1, or A2, with roughly half exhibiting the T350G polymorphism in *E6*. Univariable analysis identified significant differences between 350T and 350G intratypic variants in clinical T, N, and O staging, as well as disease-free survival. Multivariable analysis failed to identify any clinical factor as a statistically significant covariate for disease-free survival differences between 350T and 350G. Significant differences in several measures of B-cell mediated immune response were also observed between 350T and 350G intratypic variants. We suggest that HPV genetic variation may be associated with HNC clinical characteristics and may have prognostic value.

## 1. Introduction

Human papillomaviruses (HPVs) are non-enveloped viruses with a small double-stranded DNA (dsDNA) genome of about 8 kilobases in size [1]. There are over 400 HPV types that have been identified to date, with all identified types having exclusive tropism for either cutaneous or mucosal epithelia. The mucosa-associated HPVs are dichotomized as high-risk (HR) or low-risk (LR) based on their propensity to induce carcinogenesis following infection [2,3,4]. In 2018, HR HPVs were estimated to be responsible for 4.1% of the global burden of cancer, causing virtually all cases of cervical and anal cancers, a large proportion of vaginal, vulvar, and penile cancers, and a significant subset of head and neck cancers (HNCs) [5,6]. HR HPV type 16 (HPV16) is the most frequently detected HR HPV at the population level, and its high association with carcinogenesis, in comparison to other HPV types, makes HPV16 the most clinically relevant [7,8,9].

HNCs are a heterogeneous group of malignancies in the head and neck region that includes the oral cavity, oropharynx, hypopharynx, and larynx [6,10,11]. It is the 7th most diagnosed cancer worldwide, with approximately 890,000 new cases and 453,000 deaths in 2018 [5]. HR HPVs are responsible for approximately 25% of all HNCs, with 83% of the HPV-positive (HPV^+^) subtype being caused by HR HPV16 [10,12]. In comparison to the HPV-negative (HPV^−^) subtype—caused by excessive drinking and smoking—HPV^+^ HNCs are a distinct epidemiological, molecular, and clinical entity with patients exhibiting strikingly better responses to treatment and clinical outcomes [10,11,13,14,15,16,17]. Interestingly, HNCs caused by other HR HPVs other than HPV16 have been associated with different patient outcomes [18,19,20].

During infection, HPVs usurp their host cell’s DNA replication machinery to replicate their small dsDNA genome. This strategy of viral DNA replication exploits the proofreading capabilities of the host cell’s DNA polymerase, leading to a very low rate of nucleotide polymorphisms [21,22,23]. However, nucleotide polymorphisms can still arise through random mutations and can become established in a population over time or arise because of genome editing by APOBECs as part of the host’s innate immune response [24]. This genetic drift has been observed through the identification of HPV16 intratypic variants, signifying their coevolution with humans [25,26]. Notably, the *E6* oncogene from HR HPV16 is a hotspot for naturally occurring polymorphisms, whereas this seems to be rare for the *E7* oncogene [27,28,29]. The E6 oncoprotein interacts with and subsequently inhibits the activity of p53—a tumor suppressor [30]. This thwarts the triggering of p53-mediated cell cycle arrest or apoptosis upon the unsanctioned initiation of the cell cycle induced by E7 [31]. Together, these activities deregulate the cell cycle, creating an environment conducive to viral replication, which can ultimately lead to oncogenic transformation [3,32].

HPV16 is a member of the *Papillomaviridae* family of viruses, the *Alphapapillomavirus* genus, and *Alphapapillomavirus 9* species [33]. Different genera of HPVs are defined as having sequence differences of more than 40%, whereas sequence differences between 30 and 40% define HPV species [33]. Different types of HPVs have sequence differences between 10 and 30%. HPV intratypic variants have smaller genetic differences within the viral genome within a given type. Sequence differences between 1 and 10% define the main HPV16 variant lineages (A, B, C, D) and differences between 0.5 and 1% define the sublineages (A1–A4, B1–B2, D1–D3) [30]. HPV16 intratypic variants are classified into 4 major phylogenetic lineages: A (sublineages A1–A4; A1 is the reference genome/sequence), B (sublineages B1–B2), C, and D (sublineages D1–D3) [34]. Intratypic classification is based on different combinations of single nucleotide polymorphisms (SNPs) in the non-coding regulatory long control region (LCR) and/or the *E6* oncogene [34,35]. Historically, intratypic variant nomenclature was derived from the geographical origin of the populations in which they were identified [25,36]. The variants were referred to as European (A1–A3), Asian (A4), African-1 (B1–B2), African-2 (C), North American (D1), and Asian-American (D2–D3) [37,38].

In addition, there are non-lineage-specific HPV variants that have minor genetic variations that do not fit a phylogenetic tree. The prime example is HPV16 T350G which has a non-synonymous nucleotide change at position 350 of the *E6* oncogene from thymine (T) to guanine (G). This SNP at position 350 changes the original amino acid residue at position 83, within the E6 oncoprotein, from leucine (L) to valine (V) [33]. Infections with the non-lineage-specific HPV16 T350G have been associated with elevated rates of persistent infection and progression to high-grade cervical lesions [39]. Furthermore, it has been suggested that HPV16 T350G carries a higher risk for the development of cervical cancer and may be more oncogenic than the A1 reference genome [40].

Naturally occurring polymorphisms within the *E6* oncogene leading to amino acid changes could lead to alterations in the steady state levels or activity of its product, thereby having a major influence on carcinogenesis or prognosis. Alternatively, these amino acid changes could influence the antigenicity of viral-derived peptides, as demonstrated for the viral capsid proteins [41]. Perhaps the best-studied polymorphism is a T to G transversion at bp 350, which alters a leucine to valine at position 83 in the E6 oncoprotein [40,42,43,44]. Since different HPV types exhibit distinctive oncogenic potential, it is, therefore, reasonable to hypothesize that HPV16 intratypic variants also display a difference in their oncogenicity. In fact, there is compelling data that HPV16 intratypic variants influence viral persistence, progression to premalignant lesions, development of malignant lesions, and histological type of lesion in the context of cervical cancers [45,46,47,48]. Indeed, the same HPV16 intratypic variant-specific effects observed in HPV16^+^ cervical cancers could be true for HPV^+^ HNCs; however, an extensive literature search shows that this is a severely understudied area of research, particularly in North America. Due to the rarity of some of the intratypic variants in the general North American population, we combined HPV16^+^ HNC samples from a local Southwestern Ontario, Canada cohort (SWO) with those from the Cancer Genome Atlas (TCGA) HNC cohort to create a large North American cohort (CANUSA)—the increase in sample size contributes to an increase in the statistical power of this study. Then, with the large CANUSA cohort of HPV16^+^ HNC samples, we determined the distribution of intratypic variants, as well as their impact on clinical variables, tumor immune response, and patient survival outcomes.

## 2. Materials and Methods

### 2.1. Southwestern Ontario (SWO) Cohort

Approval for the study was obtained from Western University’s Ontario Research Ethics Board (LHSC HSREB #7182—9 September 2010). A retrospective search of the London Regional Cancer Program (LRCP) database was completed to identify patients diagnosed with oropharyngeal squamous cell carcinomas (OSCC) from 2003 to 2009. The following was required for patient eligibility: (1) histological confirmation of squamous cell carcinoma, (2) no prior history of head and neck cancer, and (3) the availability of a pre-treatment primary site biopsy specimen for analysis. Patient data were extracted from a retrospective chart review, which included age at diagnosis, use of tobacco and alcohol, AJCC TNM stage, treatment regimen, and post-treatment follow-up information.

After completion of cancer therapy, patients were followed at 3–6 months intervals by either a radiation oncologist or head and neck surgeon. Treatment response was evaluated by physical exam as well as computed tomography imaging as needed. All the patients were HIV-negative.

### 2.2. DNA Extraction

#### 2.2.1. Formalin-Fixed Paraffin-Embedded (FFPE) Samples

Deparaffinization and DNA extraction were performed as previously described [49]. In brief, the FFPE blocks from each patient’s primary site were sectioned and mounted on slides. The slides were then deparaffinized with washes in xylene, followed by a 1:1 xylene:ethanol mix, then ethanol twice, followed by single washes in ethanol. Lastly, the slides were washed in water for 5 min. The deparaffinized tissue was scraped into a 1.5 mL microcentrifuge tube that contained 50–100 μL of TE and proteinase K (2 mg/mL; Qiagen, Hilden, Germany) and then incubated overnight at 65 °C. Following proteinase K treatment, the samples were heated at 95 °C for 10 min and allowed to cool to room temperature.

#### 2.2.2. Fresh-Frozen (FF) Samples

DNA extraction from the FF samples was performed using the AllPrep DNA/RNA kit (Qiagen, Hilden, Germany) according to the manufacturer’s instructions. In brief, the patient tumor samples (20–30 mg) were placed in Buffer RLT Plus (Qiagen, Hilden, Germany) and homogenized with a fine-motorized tissue homogenizer (VWR 200, VWR, Radnor, Pennsylvania). The disrupted tissue was centrifuged through a QiaShredder (Qiagen, Hilden, Germany) at full speed for 3 min. The filtrate was then used to extract RNA and DNA.

### 2.3. HPV Typing, PCR, Sequencing, and Variant Identification

The HPV status and HPV type were determined with HPV type-specific primers as described previously [19,49,50]. The DNA extracted from 57 FFPE or 49 FF HPV16^+^ patient samples was used as a template to amplify the full-length HPV16 *E6* gene by PCR using 3 primer pairs as previously described (Table 1) [51]. Since formalin fixation is known to cause DNA fragmentation, we used primer pairs that generated overlapping amplicons of less than 250 base pairs [27,51]. A phusion high-fidelity PCR kit (New England Biolabs, Ipswich, MA, USA) was utilized with the following program: 98 °C for 30 s for the initial denaturation step, followed by 30 cycles that consisted of 98 °C for 5 s, 60 °C for 10 s, 72 °C for 15 s, and a final extension at 72 °C for 5 min.

The amplicons were analyzed by DNA gel electrophoresis, purified using a commercial PCR clean-up kit (GeneJET PCR Purification Kit, Thermo Scientific, Waltham, MA, USA), and Sanger sequenced by Bio Basic’s DNA sequencing service (Bio Basic, Markham, Ontario, Canada). Sequencing was performed separately with both forward and reverse primers. Only data with no discrepancies were used for analysis. Sequences were then aligned (Snapgene, San Diego, CA, USA; MUSCLE algorithm) to the reference HPV16 sequence (A1), and differences in the *E6* gene (nt: 83–559) were recorded. Samples were classified into phylogenetic branches using diagnostic SNPs in the *E6* gene as previously described (Table 2) [34,35]. It is important to note that variant lineage classification restricted to the *E6* ORF is highly correlated with those based on the HPV16 long-control region (LCR) [35].

### 2.4. The Cancer Genome Atlas (TCGA) Cohort

All the data from the Cancer Genome Atlas (TCGA) was downloaded via the Broad Genome Data Analysis Center’s Firehose server (https://gdac.broadinstitute.org/, accessed on 27 June 2018). These samples were treatment-naïve before surgical resection. The TCGA HNSC survival data sets were sourced from Liu et al. [52], with the data sets manually annotated for HPV variant status. All the patients were HIV-negative.

Variant calling was performed on the TCGA RNA-seq data using bcftools mpileup and call functions. bcftools mpileup was run with the max-depth parameter set to 10,000. bcftools call was run with the ploidy parameter set to 1. The HPV16 sublineage was established by comparing nucleotide identities (SNPs) at positions 109, 131, 132, 143, 145, 178, 276, 286, 289, 335, 350, 403, 433, and 532 within the *E6* gene [34,35].

The immune landscape features for the TCGA HNSC data set were sourced from Thorsson et al. [53]. This data included 53 immune landscape features of various predicted measures of immune infiltration by various cell types, innate and adaptive immune inflammation scores, and antigen presentation scores. The correlation of immune landscape features and HPV16^+^ variant subsets was performed via R’s built-in cor.test function, with the function being run with the linear relationship and Spearman correlation coefficient arguments. q values were calculated for each comparison group with an FDR of 10%.

### 2.5. Statistical Analysis

The HPV16 variant lineages were correlated with patient clinical variables using either Fisher’s exact test or Freeman–Halton extension of Fisher’s exact test (Fisher–Freeman–Halton test). Five-year overall and disease-free survival outcomes were compared to the HPV16 variants as indicated. Log-rank statistical analysis was performed using GraphPad Prism v7.0 (Graphpad Software, Inc., San Diego, CA, USA). The figures were assembled using Adobe Illustrator 2023 (Adobe Systems Inc., San Jose, CA, USA). The univariate analysis was performed for the indicated HPV16 variants and the following clinical variables: age, sex, subsite, T stage, N stage, overall stage, smoking status, smoking frequency, and treatment, through RStudio (version 1.2.1335) based on a Cox proportional-hazards model with the survival package (version 2.41-3). In addition, Multivariate analyses were performed using a stepwise bidirectional method. The smoking frequency clinical variable was stratified as heavy smokers (>20 pack year history), light smokers (≤20 pack year history), or non-smokers. The statistical *p* values were derived from the Wald test on hazard ratios.

## 3. Results

### 3.1. Study Cohorts and Distribution of HPV16 Intratypic Variants in HNCs from North America

Utilizing our local Southwestern Ontario, Canada cohort (SWO) of HPV16^+^ HNC patient samples, we PCR amplified extracted DNA, subjected the amplified DNA to Sanger sequencing, and aligned the sequencing results to the reference HPV16 sequence A1. Of the 94 samples that were analyzed from the SWO cohort, 38 were classified into the A1 sublineage, 54 into the A2 sublineage, and 2 into the D2/D3 sublineage (Table 3).

Next, we utilized whole-genome sequencing (WGS) data from a secondary cohort of 55 HPV16^+^ HNC patient samples from the Cancer Genome Atlas (TCGA), which were collected from patients in Canada and the USA [54]. Similarly, the HPV16 genomes were classified into sublineages based on combinations of SNPs in the viral *E6* oncogene. Of the 55 samples analyzed from this second cohort, 20 were classified into the A1 sublineage, 20 into A2, 4 into A4, 3 into B1, 1 into B2, 1 into C, and 6 into D2/D3 (Table 3). In addition, we also analyzed the *E6* oncogene in both cohorts for a SNP at position 350. This is the most frequent SNP in HPV16 and leads to a leucine-to-valine change at residue 83 in the E6 oncoprotein. This SNP has been reported to alter the oncogenic properties of E6 and has been correlated with disease outcomes in cervical cancer [40,41,42,43]. The SWO cohort had 33 samples that were classified as 350T and 40 that were 350G. Whereas the TCGA cohort had 16 samples that were classified as 350T and 9 that were 350G (Table 3).

To increase the statistical power of our study and account for the rarity of some of the intratypic variants in the populations of Canada and the United States, we increased the sample size of our cohort by combining both the SWO and TCGA cohorts (CANUSA). This new cohort now contained 149 HPV16^+^ HNC samples of which 58 were classified into the A1 sublineage, 74 into A2, 4 into A4, 3 into B1, 1 into B2, 1 into C, and 8 into D2/D3 (Table 3). Furthermore, 49 samples were classified as 350T, and 49 were 350G.

**Figure 1:**
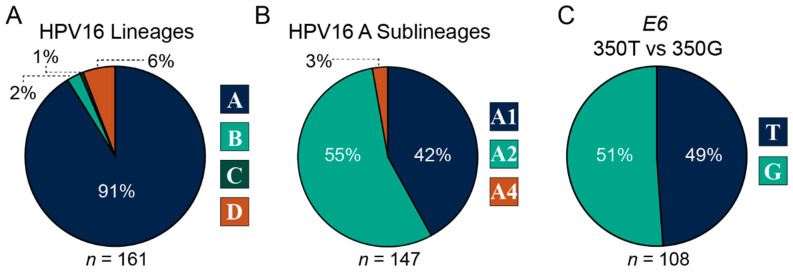
Distribution of HPV16 intratypic variants. Pie charts depicting the distribution of HPV16 lineages (**A**), HPV16 A sublineages (**B**), and single nucleotide polymorphism at position 350 of the HPV16 oncogene *E6* (**C**). The numbers below charts (*n*) indicate total numbers per analysis.

When analyzing the distribution of HPV16 intratypic variants in HNCs, we also included samples that had clinical data missing from the SWO cohort (Table 3). This increased the size of the CANUSA cohort from 149 samples to a total of 161 samples with variant calls that were classified into the 4 major phylogenetic lineages A, B, C, and D (Figure 1). The distribution of the major lineages in the CANUSA cohort was 91% A, 2% B, 1% C, and 6% D (Figure 1A). Since most of the samples were classified into lineage A, we specifically analyzed the distribution of sublineages within lineage A. The distribution of the A sublineages was 42% A1 (reference genome), 55% A2, and 3% A4 (Figure 1B). Notably, there were no samples that were classified into the A3 sublineage. Finally, we analyzed the distribution of samples that contained either thymine (T) at position 350 (reference genome) or the guanine (G) SNP at position 350. The distribution was 49% 350T and 51% 350G (Figure 1C). Taken together, the CANUSA cohort is predominantly made up of the A lineage, specifically the sublineages A1 and A2. Furthermore, roughly the same number of samples were segregated as 350T or 350G.

### 3.2. Impact of HPV16 Intratypic Variants in HNCs on Clinical Variables

Next, we wanted to determine the association of HPV16 intratypic variants with various clinical variables that include age, sex, smoking history, anatomical subsite of the cancer, T stage, N stage, and O stage. Since most of the samples in the CANUSA cohort were classified into major lineage A, we were unable to assess the association of clinical variables between all 4 major lineages. We first assessed the impact of lineage A vs. lineages B/C/D on clinical variables and found no statistically meaningful associations (Table S1A). We then assessed lineage A vs. lineage B vs. lineage D and the results indicated that there were no statistically meaningful associations with those comparisons (Table S1B). A comparison of the impact of lineage A vs. lineage D on clinical variables detected no statistically significant results (Table S1C).

Since most of the samples are predominantly classified into the A sublineage, we analyzed the association of clinical variables with sublineages A1, A2, and A4. Our results showed no significant association with any of the clinical variables analyzed (Table S1D). However, when we analyzed the impact of sublineage A1 vs. sublineage A2 on clinical variables, the association with the sublineages and O stage trended towards significance (*p* = 0.05544), and smoking status was significant (*p* = 0.029; Table 4). Finally, we focused on the SNP at position 350 of the *E6* viral ORF and analyzed the association of 350T (reference genome) vs. 350G on clinical variables. The results showed a statistically significant association with T stage (*p* = 0.01129), N stage (*p* = 0.004325), O stage (*p* = 0.001462), and smoking status (*p* = 0.017; Table 5). Specifically, more patients with the 350T HPV16 genome (reference genome) were staged with T4, while more with the 350G genome were staged into T3. Similarly, there were more patients with the 350T HPV16 reference genome that were staged with N0 and N3 compared to those with 350G that were more frequently staged at N1. Notably, there were no patients with the 350G HPV16 intratypic variant that had O stage I or II. Taken together, this data suggests that specific intratypic HPV16 variants may impact clinical variables and identify significant associations between SNPs in the *E6* viral oncogene and the clinical parameters analyzed.

### 3.3. Impact of HPV16 Intratypic Variants on Clinical Outcomes

There is evidence, in the context of cervical cancer (CC), that HPV16 intratypic variants can influence virus persistence, infection recurrence, disease risk, and cancer survival [43,55,56,57]. To determine if those intratypic-specific influences hold true for HPV16^+^ HNCs, we grouped the CANUSA cohort by lineages, sublineages, and SNP present at position 350 of the viral *E6* oncogene and correlated those groups with overall and disease-free survival (Figures S1 and S2).

**Figure 2:**
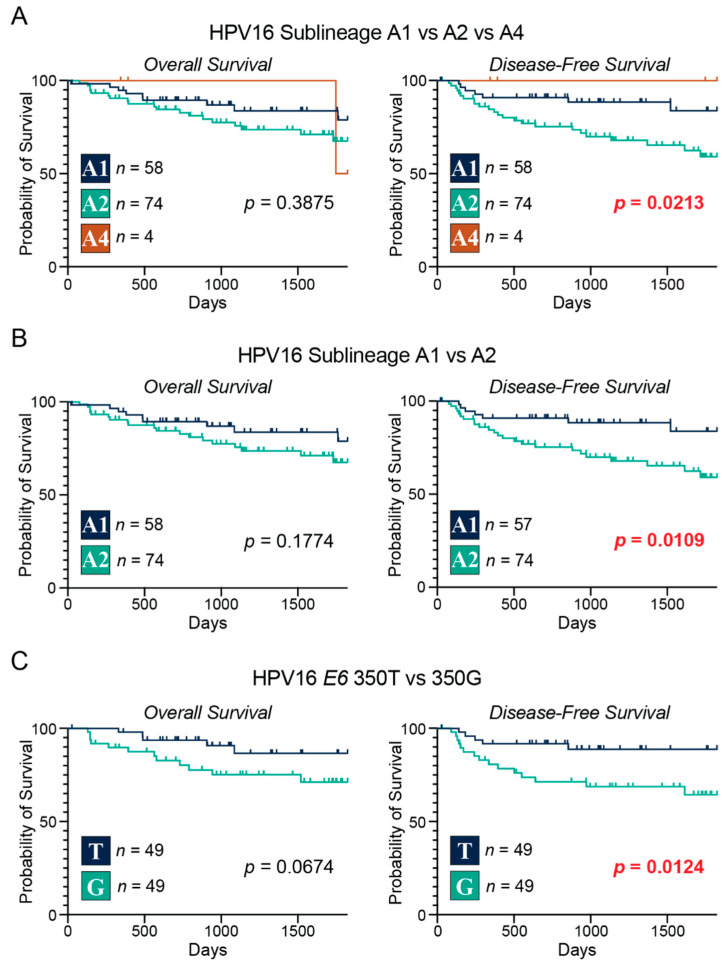
Correlation of HPV16 intratypic variants with overall and disease-free survival in patients with head and neck cancers. Overall and disease-free survival of patients grouped by HPV16 sublineages A1, A2, and A4 (**A**); sublineages A1 and A2 (**B**); HPV16 *E6* 350T and 350G single nucleotide polymorphism (**C**). Comparison between groups was calculated with the 2-sided log-rank test (*p*-value). The number of samples included in each group is indicated with *n*. *p* ≤ 0.05 are in red.

We began our analysis by correlating the major lineages with overall and disease-free survival. The results from lineage A vs. lineages B/C/D yielded no significant survival differences between the 2 groups (Figure S1A). Likewise, the correlations with survival between lineage A vs. lineage B vs. lineage D (Figure S1B) or lineage A vs. lineage D (Figure S1C) were also non-significant. We then focused on the sublineages of lineage A and correlated them to our survival metrics. When comparing sublineage A1 to A4 (Figure S1D) or sublineage A2 to A4 (Figure S1E) there were no statistically significant correlations with survival. However, when we compared sublineages A1 vs. A2 vs. A4 (Figure 2A) there was a significant correlation with disease-free survival (*p* = 0.0213). We then did a pairwise comparison between sublineage A1 and A2 and found that those patients with the A1 sublineage of HPV16 had a significant correlation with disease-free survival (*p* = 0.0109; Figure 2B). Finally, we compared patients with the HPV16 genomes that had the SNP at position 350 of the *E6* viral oncogene and correlated the association of 350T vs. 350G with survival (Figure 2C). The results indicated that those patients with the 350T genome (reference genome) had statistically better disease-free survival compared to their 350G counterparts (*p* = 0.0124).

Results from univariable Cox proportional hazards regression analysis similarly identified 350G as a significant risk factor for disease-free survival (HR = 3.55, *p* = 0.030), but not overall survival (Table 6 and Table 7). The multivariable analysis did not identify any clinical feature or 350G as statistically significant independent predictors of worse disease-free survival. Although not significant, 350G (HR = 2.78, *p* = 0.090), along with age (HR = 1.03, *p* = 0.144), and T-stage (HR = 2.86, *p* = 0.082) were the top three covariates in the predicted model of survival (Table 7). Univariable and multivariable analysis did not identify any significant correlations between sublineage A1 vs. A2 for either disease-free or overall survival (Tables S2A,B), although A2 trended towards worse disease-free survival (HR = 2.52, *p* = 0.054) and was identified as a covariate in that model. Taken together, these results show that there are differences in disease-free survival between patients that harbor different A sublineages of the HPV16 genome, as well as those with HPV16 genomes that had a SNP at position 350 of the *E6* viral oncogene.

### 3.4. Impact of HPV16 Intratypic Variants on Immune Characteristics

**Figure 3:**
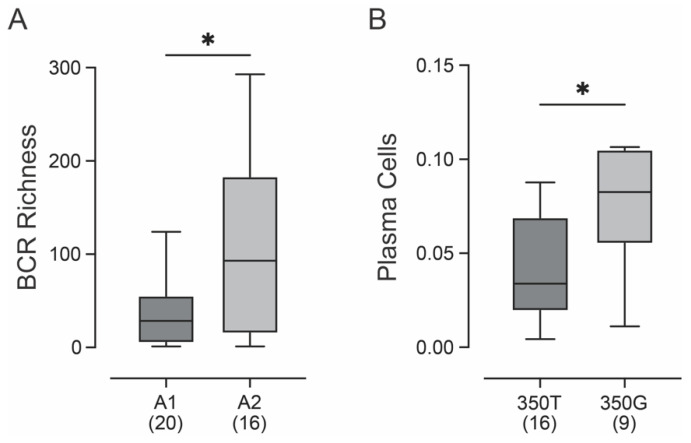
Correlation of HPV16 intratypic variants with immune characteristics of patients with head and neck cancer. Predicted B-cell receptor (BCR) richness was significantly increased in HPV16^+^ HNC associated with A2 vs. A1 sublineage (**A**). Predicted plasma cell presence in HPV16^+^ HNC expressing the T350G polymorphism was significantly increased (**B**). Comparison between groups was performed with a two-tailed non-parametric Mann–Whitney U test. The number of samples included in each group is indicated in brackets. * *p* ≤ 0.05.

Recent breakthroughs in cancer immunotherapy have clearly demonstrated the critical role of the immune system in controlling malignancy. Thorsson et al. [53] recently calculated 53 distinct immune signatures related to the tumor immune microenvironment for all the TCGA samples with RNA-seq data. These include various predicted measures of immune infiltration by various cell types, innate and adaptive immune inflammation scores, and antigen presentation scores. We used this data to look for potential differences in the tumor immune microenvironment between the A1 and A2 sublineage samples from the TCGA head and neck cancer cohort, as no RNA-seq data is available for the SWO cohort. Of the wide variety of immune signatures, only B-cell receptor (BCR) richness was significantly different between these two sublineages after false-discovery correction (Figure 3A). This measurement reflects the relative abundance of unique clones of BCRs, which was significantly higher in A2 samples compared to A1 samples. The increased diversification of the BCR suggests that there may be minor differences in the immune response in HNCs caused by these two sublineages. We similarly compared the samples stratified as 350T or 350G and identified an increase in plasma cells, which are terminally differentiated cells that arise from antigenically stimulated B lymphocytes, in those samples with the 350G polymorphism (Figure 3B).

## 4. Discussion

HPV16 is responsible for an estimated 83% of all HPV^+^ head and neck cancers, and 50–60% of CCs [10,12,58]. The most predominant HPV16 variant lineage in CC is the reference/prototype A1. Notably, studies have revealed that the non-A variant lineages of HPV16 are associated with a higher risk of precancerous lesions and development of CC [34,59], or associated with higher rates of persistent infection and progression to CC when compared to the other lineage variants [59,60,61]. In addition, the well-characterized HPV16 T350G, which leads to the L83V E6 substitution, has been associated with higher rates of persistent infection and progression to cervical cancers [39,44,45,62,63]. In the context of HNCs, the same HPV16 variant lineage-specific effects observed in HPV16^+^ cervical cancers could be true; however, few studies reporting on the distribution of HPV16 variant lineages in HNC or the non-lineage-specific HPV16 T350G were available [37].

In this study, we combined our local cohort of HPV16^+^ HNC samples from Southwestern Ontario, Canada (SWO) with those from the Cancer Genome Atlas (TCGA) HPV16^+^ HNC cohort to create a larger North American cohort (CANUSA). This cohort, representative of Canada and the United States of America, increases the statistical power of the study and increases the diversity of the samples analyzed (Table 3). Utilizing our CANUSA cohort of 161 HPV16^+^ HNC samples—only 149 samples had clinical data available—we determined the distribution of variant lineages, their association with patient clinical variables, and their impact on patient survival outcomes.

The distribution of HPV16 intratypic variant lineages in HNCs was 91% A, 2% B, 1% C, and 6% D (Figure 1A). Furthermore, within the A lineage, the distribution was 42% A1 (reference/prototype sequence), 55% A2, and 3% A4 (Figure 1B). When analyzing the distribution of non-lineage-specific HPV16 T350G, we found that 49% of the samples were classified as 350T (reference/prototype sequence), whereas 51% were 350G (Figure 1C). These results show that the CANUSA cohort is predominately made up of variant lineage A, specifically sublineages A1 and A2, and the non-lineage-specific variant T350G is present in roughly the same number of samples compared to the reference/prototype genome. These results are very similar to recent studies from various sites in the USA (Table 8) [64,65,66], although the study from the University of North Carolina with 107 HNC samples differed in that it reported a much larger fraction of the A lineage were A1 (85%), with only 10% A2 [64].

When we analyzed the association of sublineages A1 or A2 with patient clinical variables, the association with the sublineages and O stage was trending towards significance (*p* = 0.05544), and smoking status was significant (*p* = 0.029; Table 4). When we focused on the non-lineage-specific variant T350G compared to the reference/prototype genome, the results showed a statistically significant association with T stage (*p* = 0.01129), N stage (*p* = 0.004325), O stage (*p* = 0.001462), and smoking status (*p* = 0.017; Table 5). Specifically, there were more patients with the reference/prototype genome that were staged with T4, N0, or N3, whereas more patients with the T350G variant were staged into T3 or N1. These results identified a significant association between SNPs in the *E6* viral oncogene and the clinical parameters analyzed, illustrating the association of HPV16 intratypic variants on HNC patient clinical factors.

When we correlated the HPV16 sublineages of lineage A with overall survival and disease-free survival, we observed that patients with the A1 reference/prototype genome of HPV16 had significantly improved disease-free survival compared to their A2 counterparts (Figure 2). Although not directly comparable, a smaller study reported that sublineage A1 exhibited worse relapse-free survival compared to the collective aggregation of all other sublineages [64]. No direct comparison between sublineage A1 and A2 was performed in that cohort, as there were relatively few A2 samples.

In addition to survival differences based on sublineage, when correlating the non-lineage-specific variant T350G and the reference/prototype genome (350T) with survival, we found that those patients with the reference/prototype genome (350T) had significantly greater disease-free survival (*p* = 0.0124; Figure 2C, Table 7). No changes in overall survival were noted, likely related to the low level of mortality in this cohort. These results suggest that even minor nucleic acid changes in the coding region of the *E6* oncogene can impact patient survival outcomes. A larger cohort study using 384 HPV16^+^ HNC samples from several centers in the USA assessed genetic variation across the entire viral genome for correlation with overall survival. While a few SNPs were associated with overall survival, T350G was not, and this study did not report on disease-free survival or compare sublineages directly for patient outcomes [65].

Given the critical role of the immune system in controlling malignancy, we looked for differences in immune signatures that reflect differences in the tumor immune microenvironment between the A1 and A2 sublineage samples or 350T and 350G samples from the TCGA. After false-discovery correction, only B-cell receptor (BCR) richness was significantly different between the A1 and A2 sublineages (Figure 3A) and plasma cell abundance between 350T and 350G (Figure 3B). The increased diversification of the BCR is consistent with differences in the immune response in HNCs caused by these two sublineages. We similarly compared the samples stratified as 350T or 350G and identified an increase in plasma cells in those samples with the 350G polymorphism (Figure 3B). B-cells are important players in immune responses to cancer and differences in B-cell infiltration in HPV^+^ and HPV^−^ HNC have been reported [67]. Additionally, HPV antigen-specific activated and germinal center B cells, as well as plasma cells can be found in the HPV16^+^ HNC tumor microenvironment [68], the differences we identified in B-cell richness and plasma cell frequency could contribute mechanistically to altered patient outcomes associated with HPV variants.

In conclusion, our findings suggest that minor sequence variations within HPV16 appear to be associated with HPV^+^ HNC patient characteristics and prognosis. Even straightforward targeted sequencing of small portions of the HPV genome may be sufficient to obtain clinically relevant information that can help stratify patient risk. However, this study was limited by the number of available sequences, and powering future investigations with much larger cohort sizes will be necessary to unequivocally establish if intratypic variant typing is of prognostic value for HNC.

## Figures and Tables

**Table 1:** Primers used for the amplification of HPV16 *E6*.

Primers	Sequences (5′–3′)	Position (nt)	Product Size (bp)
E6-1 Forward	TTGAACCGAAACCGGTTAGT	46–65	211
E6-1 Reverse	GCATAAATCCCGAAAAGCAA	237–256	
E6-2 Forward	GCAACAGTTACTGCGACGTG	204–224	235
E6-2 Reverse	GGACACAGTGGCTTTTGACA	419–438	
E6-3 Forward	CAGCAATACAACAAACCGTTG	371–391	220
E6-3 Reverse	TCATGCAATGTAGGTGTATCTCC	568–590	

nt = nucleotides; bp = base pairs.

**Table 2:** HPV16 lineage reference sequences and diagnostic SNPs within *E6*.

Lineage	Sublineage	Variant Genome ID	GenBank Accession Numbers	*E6* Nucleotide Position													
				1 0 9	1 3 1	1 3 2	1 4 3	1 4 5	1 7 8	2 7 6	2 8 6	2 8 9	3 3 5	3 5 0	4 0 3	4 3 3	5 3 2
A	A1	Ref	K02718	T	A	G	C	G	T	A	T	A	C	T	A	G	A
	A2	W0122	AF536179	—	—/G	—	—	—	—	—	—	—	—	—/G	—	—	—
	A3	AS411	HQ644236	—	—	—	—	—	—	G	—	—	—	—	—	—	—
	A4	W0724	AF534061	—	—	—	—	—	G/A	—	—	—	—	—	—	—	—
B	B1	W0236	AF536180	—	—	C	G	T	—	—	A	G	T	—	—	—	—
	B2	Z109	HQ644298	—	G	—	G	T	—	—	A	G	T	—	—	—	—
C		R460	AF472509	C	—	T	G	T	—	—	A	G	T	—	G	—	—
D	D1	QV00512	HQ644257	—	—	—	—	T	—	—	A	G	T	G	—	—	—
	D2	QV15321	AY686579	—	—	—	—	T	—	—	A	G	T	G	—	—	G
	D3	QV00995	AF402678	—	—	—	—	T	—	—	A	G	T	G	—	A	G

SNP = single nucleotide polymorphism.

**Table 3:** Clinical patient variables and cohort comparisons.

Clinical Variables	SWO Cohort	TCGA Cohort	CANUSA Cohort
	*n* = 94 ^a^	*n* = 55	*n* = 149
**Age at diagnosis**			
Median (IQR)	57 (52–66)	57 (50.5–61)	57 (50.5–66)
**Sex**			
Female	15	6	21
Male	79	49	128
**Subsite**			
Tonsil	61	30	91
BOT	28	10	38
Other	5	15	20
**T Stage ^b^**			
T1	14	5	19
T2	36	28	64
T3	17	9	26
T4	19	12	31
**N Stage ^c^**			
N0	10	15	25
N1	14	4	18
N2	54	33	87
N3	8	2	10
**O Stage ^d^**			
I	0	1	1
II	6	9	15
III	11	6	17
IV	58	39	97
**Smoking Status**			
Never	28	19	47
Former	26	26	52
Current	40	10	50
**Smoking Frequency ^e^**			
Non-Smoker	28	19	47
Light Smoker	22	10	32
Heavy Smoker	39	20	59
**HPV16 Sublineages**			
A1	38	20	58
A2	54	20	74
A3	0	0	0
A4	0	4	4
B1	0	3	3
B2	0	1	1
C	0	1	1
D1	0	0	0
D2/D3	2	6	8
D3	0	0	0
**HPV16 *E6***			
350T	33	16	49
350G	40	9	49

SWO = Southwestern Ontario; TCGA = The Cancer Genome Atlas; CANUSA = Canada–USA; IQR = interquartile range; BOT = base-of-tongue; HPV16 = human papillomavirus type 16. ^a^ Clinical data was available for 94/106 samples with variant calls. ^b^ T stage data missing for 8 patients from the SWO cohort and undefined for 1 patient from the TCGA cohort. ^c^ N stage data missing for 8 patients from the SWO cohort and 1 patient from the TCGA cohort. ^d^ O stage data missing for 19 patients from the SWO cohort. ^e^ Smoking frequency data missing for 5 patients from the SWO cohort and 6 patients from the TCGA cohort.

**Table 4:** Association of clinical variables with HPV16 sublineages A1 and A2.

Clinical Variables		Sublineage A1	Sublineage A2	Total	*p* Value
**Age**	≤60	41	43	84	0.149
	>60	17	31	48	
**Sex**	Female	10	10	20	0.628
	Male	48	64	112	
**Subsite**	Tonsil	34	46	80	0.907
	BOT	16	18	34	
	Other	8	10	18	
**T Stage**	T1	8	9	17	0.210
	T2	27	28	55	
	T3	6	17	23	
	T4	15	13	28	
**N Stage**	N0	11	11	22	0.165
	N1	4	14	18	
	N2	35	38	73	
	N3	6	4	10	
**O Stage**	I	1	0	1	0.055
	II	9	4	13	
	III	4	12	16	
	IV	41	42	83	
**Smoking Status**	Never	24	19	43	**0.029**
	Former	20	21	41	
	Current	14	34	48	
**Smoking Frequency**	Non-Smoker	24	19	43	0.089
	Light Smoker	11	14	25	
	Heavy Smoker	18	36	54	

BOT = base-of-tongue. *p* ≤ 0.05 are in red.

**Table 5:** Association of clinical variables with HPV16 *E6* 350T and 350G single nucleotide polymorphism.

Clinical Variables		*E6* 350T	*E6* 350G	Total	*p* Value
**Age**	≤60	34	27	61	0.211
	>60	15	22	37	
**Sex**	Female	9	7	16	0.785
	Male	40	42	82	
**Subsite**	Tonsil	28	31	59	0.840
	BOT	14	12	26	
	Other	7	6	13	
**T Stage**	T1	7	7	14	**0.011**
	T2	22	15	37	
	T3	4	16	20	
	T4	14	7	21	
**N Stage**	N0	8	3	11	**0.004**
	N1	2	13	15	
	N2	31	27	58	
	N3	6	2	8	
**O Stage**	I	1	0	1	**0.001**
	II	6	0	6	
	III	2	10	12	
	IV	37	28	65	
**Smoking Status**	Never	20	15	35	**0.017**
	Former	20	12	32	
	Current	9	22	31	
**Smoking Frequency**	Non-Smoker	20	15	35	0.346
	Light Smoker	10	9	19	
	Heavy Smoker	15	22	37	

BOT = base-of-tongue. *p* ≤ 0.05 are in red.

**Table 6:** Univariable and multivariable Cox proportional hazards regression for overall survival with HPV16^+^ 350T and 350G in the CANUSA cohort.

Variable	Univariable Analysis		Multivariable Analysis	
	HR (95% CI)	*p* Value	HR (95% CI)	*p* Value
**Age at diagnosis**	1.02 (0.97–1.07)	0.423		
**Sex**				
Male vs. Female	0.41 (0.12–1.32)	0.134		
**Subsite**				
Tonsil vs. base-of-tongue	0.74 (0.22–2.52)	0.629		
Other vs. base-of-tongue	1.06 (0.19–5.77)	0.950		
**T stage**				
T3–T4 vs. T1–T2	4.62 (1.27–16.8)	**0.020**		
**N stage**				
N2–N3 vs. N0–N1	0.43 (0.14–1.27)	0.126		
**Overall Stage**				
III–IV vs. I–II	7.59 × 10^7^ (0–Inf)	0.998	4.62 (1.27–16.8)	**0.020**
**Smoking status**				
Former vs. Never	0.47 (0.09–2.40)	0.360		
Current vs. Never	1.35 (0.41–4.45)	0.620		
**Smoking Frequency**				
Light vs. Never	0.66 (0.13–3.40)	0.619		
Heavy vs. Never	1.05 (0.32–3.45)	0.938		
**HPV16 T350G**				
350G vs. 350T	2.91 (0.89–9.46)	0.076		
**Treatment**				
Chemotherapy + Radiation	8.98 × 10^−1^ (0.27–2.95)	0.859		
Surgery + Chemotherapy + Radiation	1.29 × 10^−8^ (0–Inf)	0.999		
Surgery + Radiation	1.30 × 10^−8^ (0–Inf)	0.999		
Surgery Alone	1.11 (0.34–3.66)	0.859		
Radiation Alone	1.29 × 10^−8^ (0–Inf)	0.999		

*p* ≤ 0.05 are in red.

**Table 7:** Univariable and multivariable Cox proportional hazards regression for disease-free survival with HPV16^+^ 350T and 350G in the CANUSA cohort.

Variable	Univariable Analysis		Multivariable Analysis	
	HR (95% CI)	*p* Value	HR (95% CI)	*p* Value
**Age at diagnosis**	1.04 (0.99–1.08)	0.124	1.03 (0.99–1.08)	0.144
**Sex**				
Male vs. Female	0.47 (0.15–1.48)	0.198		
**Subsite**				
Tonsil vs. base-of-tongue	0.70 (0.23–2.14)	0.530		
Other vs. base-of-tongue	0.89 (0.17–4.58)	0.888		
**T stage**				
T3–T4 vs. T1–T2	3.86 (1.23–12.14)	**0.021**	2.86 (0.88–9.35)	0.082
**N stage**				
N2–N3 vs. N0–N1	0.74 (0.25–2.17)	0.586		
**Overall Stage**				
III–IV vs. I–II	7.63 × 10^7^ (0–Inf)	0.998		
**Smoking status**				
Former vs. Never	0.79 (0.22–2.80)	0.712		
Current vs. Never	0.98 (0.30–3.21)	0.968		
**Smoking Frequency**				
Light vs. Never	0.84 (0.21–3.35)	0.799		
Heavy vs. Never	0.91 (0.29–2.82)	0.867		
**HPV16 T350G**				
350G vs. 350T	3.55 (1.13–11.17)	**0.030**	2.78 (0.85–9.05)	0.090
**Treatment**				
Chemotherapy + Radiation	6.17 × 10^−1^ (0.22–1.77)	0.369		
Surgery + Chemotherapy + Radiation	1.38 × 10^−8^ (0–Inf)	0.998		
Surgery + Radiation	1.42 × 10^−8^ (0–Inf)	0.999		
Surgery Alone	1.62 (0.57–4.64)	0.369		
Radiation Alone	1.41 × 10^−8^ (0–Inf)	0.999		

*p* ≤ 0.05 are in red.

**Table 8:** The distribution of HPV16 intratypic variants from recent studies in the USA.

	Study	CANUSA		Vanderbilt [66]		North Carolina [64]		Vanderbilt/Pittsburgh [65]	
**HPV16 Variant**	A	136 (91.3%)	A1: 58 (42.6%)	191 (90.1%)	A1: 112 (58.33%)	91 (85%)	A1: 77 (84.6%)	347 (90.4%)	A1: 215 (62%)
			A2: 74 (54.4)		A2: 63 (32.8%)		A2: 9 (9.89%)		A2: 107 (30.8%)
			A3: 0 (0%)		A3: 3 (1.56%)		A3: 3 (3.3%)		A3: 3 (0.865%)
			A4: 4 (2.94%)		A4: 14 (7.29%)		A4: 2 (2.2%)		A4: 22 (6.34%)
	B	4 (2.68%)		1 (0.472%)		1 (0.934%)		1 (0.26%)	
	C	1 (0.671%)		4 (1.89%)		2 (1.87%)		6 (1.56%)	
	D	8 (5.37%)		16 (7.55%)		13 (12.1%)		30 (7.81%)	
	Total	149 (100%)	136 (100%)	212 (100%)	192 (100%)	107 (100%)	91 (100%)	384 (100%)	347 (100%)

## Data Availability

Data are contained within the article and Supplementary Materials.

## Supplementary Materials

The following supporting information can be downloaded at: https://www.mdpi.com/article/10.3390/v15122411/s1, Table S1. Association of clinical variables with (A): HPV16 lineages A and B/C/D, (B): HPV16 lineages A, B, and D, (C): HPV16 lineages A and D, (D): HPV16 sublineages A1, A2, and A4; Table S2. Univariable and multivariable Cox proportional hazards regression for (A) overall survival with HPV16+ A1 and A2 in the CANUSA cohort. (B) disease-free survival with HPV16+ A1 and A2 in the CANUSA cohort.
