# Supplementary material for: HPV16 Intratypic Variants in Head and Neck Cancers: A North American Perspective
Source: Viruses. 2023 Dec 12;15(12):2411. doi: 10.3390/v15122411 (PMC10747106; doi:10.3390/v15122411)
Supplement: Supplementary file 1 [file viruses-15-02411-s001.zip › Supplementary Table S2.pdf]

Table S2A. Univariable and multivariable Cox proportional hazards regression for overall survival with HPV16+ A1 and A2 sublineages in the CANUSA cohort.

Variable	Univariable Analysis HR (95% CI)	Univariable Analysis *p* value	Multivariable Analysis HR (95% CI)	Multivariable Analysis *p* value
**Age at diagnosis**	1.02 (0.99 – 1.06)	0.200		
**Sex:**				
Male vs. Female	0.38 (0.16 – 0.94)	**0.036**	0.49 (0.20 – 1.22)	0.126
**Subsite:**				
Tonsil vs. base-of-tongue	0.74 (0.28 – 1.96)	0.540		
Other vs. base-of-tongue	1.32 (0.37 – 4.68)	0.669		
**T stage:**				
T3 – T4 vs. T1 – T2	3.28 (1.34 – 8.05)	**0.009**	2.82 (1.13 – 7.01)	**0.026**
**N stage:**				
N2 – N3 vs. N0 – N1	0.43 (0.19 – 0.99)	**0.049**	0.50 (0.22 – 1.17)	0.109
**Overall Stage:**				
III – IV vs. I – II	0.99 (0.23 – 4.26)	0.991		
**Smoking status:**				
Former vs. Never	0.85 (0.27 – 2.68)	0.782		
Current vs. Never	1.18 (0.45 – 3.13)	0.732		
**Smoking Frequency:**				
Light vs. Never	0.68 (0.18 – 2.65)	0.583		
Heavy vs. Never	1.21 (0.47 – 3.07)	0.693		
**HPV16 A Sublineages:**				
A2 vs A1	1.70 (0.71 – 4.06)	0.231		
**Treatment:**				
Chemotherapy + Radiation	6.08e-01 (0.26 – 1.43)	0.253		
Surgery + Chemotherapy + Radiation	1.39e-08 (0 – Inf)	0.998		
Surgery + Radiation	1.43e-08 (0 – Inf)	0.999		
Surgery Alone	1.64 (0.70 – 3.85)	0.253		
Radiation Alone	1.43e-08 (0 – Inf)	0.998		

Table S2B. Univariable and multivariable Cox proportional hazards regression for disease-free survival with HPV16+ A1 and A2 sublineages in the CANUSA cohort.

Variable	Univariable Analysis HR (95% CI)	Univariable Analysis *p* value	Multivariable Analysis HR (95% CI)	Multivariable Analysis *p* value
**Age at diagnosis**	1.04 (1.00 – 1.08)	**0.042**	1.03 (0.99 – 1.08)	0.068
**Sex:**				
Male vs. Female	0.45 (0.18 – 1.15)	0.095	2.99 (0.11 – 0.84)	**0.021**
**Subsite:**				
Tonsil vs. base-of-tongue	0.60 (0.23 – 1.55)	0.292		
Other vs. base-of-tongue	1.18 (0.34 – 4.02)	0.798		
**T stage:**				
T3 – T4 vs. T1 – T2	3.34 (1.36 – 8.21)	**0.008**	2.34 (0.91 – 6.03)	0.077
**N stage:**				
N2 – N3 vs. N0 – N1	0.76 (0.31 – 1.81)	0.536		
**Overall Stage:**				
III – IV vs. I – II	2.05 (0.27 – 15.35)	0.483		
**Smoking status:**				
Former vs. Never	1.24 (0.43 – 3.56)	0.687		

Current vs. Never	0.98 (0.35 – 2.70)	0.965		
**Smoking Frequency:**				
Light vs. Never	0.72 (0.19 – 2.78)	0.630		
Heavy vs. Never	1.24 (0.49 – 3.16)	0.646		
**HPV16 A Sublineages:**				
A2 vs A1	2.52 (0.99 – 6.44)	0.054	2.00 (0.76 – 5.26)	0.158
**Treatment:**				
Chemotherapy + Radiation	3.75e-01 (0.16 – 0.90)	**0.029**	3.99e-01 (0.17 – 0.96)	**0.041**
Surgery + Chemotherapy + Radiation	1.51e-08 (0 – Inf)	0.998		
Surgery + Radiation	1.59e-08 (0 – Inf)	0.999		
Surgery Alone	2.67 (1.11 – 6.42)	**0.029**		
Radiation Alone	1.59e-08 (0 – Inf)	0.998		
